# Molecular Simulations of Adsorption and Energy Storage of R1234yf, R1234ze(z), R134a, R32, and their Mixtures in M-MOF-74 (M = Mg, Ni) Nanoparticles

**DOI:** 10.1038/s41598-020-64187-x

**Published:** 2020-04-29

**Authors:** Shouyin Cai, Sen Tian, Yuyi Lu, Guangjin Wang, Yu Pu, Kang Peng

**Affiliations:** 1 Key Laboratory of Low-grade Energy Utilization Technologies & Systems, Ministry of Education, College of Energy and Power Engineering, Chongqing University, Chongqing, 400044 P.R. China; 2 State Key Laboratory of Coal Mine Disaster Dynamics and Control, School of Resources and Safety Engineering, Chongqing University, Chongqing, 400044 P.R. China; 3 Yunnan Key Laboratory of Sino-German Blue Mining and Utilization of Special Underground Space, Faculty of Land Resources Engineering, Kunming University of Science and Technology, Kunming, 650093 P.R. China

**Keywords:** Energy storage, Nanoparticles, Nanoparticles

## Abstract

The refrigerant circulation heat can be enhanced through the mutual transformation between thermal energy and surface energy during the adsorption and separation process of fluid molecules in porous materials. In this paper, the adsorption and energy storage of R1234ze(z), R1234yf, R32 and R134a, as well as their mixed refrigerants in Mg-MOF-74 and Ni-MOF-74 nanoparticles were investigated by means of molecular dynamics simulations and grand canonical Monte Carlo simulations. The results suggested that, in the case of pure refrigerant adsorption, the adsorption quantities of R32 and R134a in MOFs were higher than those of R1234yf and R1234ze(z). However, in the case of saturation adsorption, the desorption heat of R32 was lower than that of R1234yf and R1234ze(z). The addition of MOF-74 nanoparticles (NPs) could enhance the energy storage capacity of the pure refrigerant; besides, R1234yf and R1234ze(z) nanofluids had superior enhancement effect to that of R32 nanofluid. In mixed refrigerant adsorption, the adsorption quantities of R1234ze(z) and R1234yf were lower than those of R32 and R134a; with the increase in temperature, the adsorption of R1234ze(z) and R1234yf showed a gradually increasing trend, while that of R32 was gradually decreased.

## Introduction

With the persistent growth in the global population and the rapid development of economy, the significance to the development and utilization of energy has become increasingly prominent. Enhancing the efficiency of energy use is an effective way to relieve the energy crisis. Actually, there are large amounts of low grade energy urgently to be recovered in the nature and industry, such as solar energy, geothermal energy and industrial waste heat^1,2^. Typically, Organic Rankine Cycle (ORC) that utilizes the refrigerant as the working fluid is one of the effective ways to utilize the low grade energy. And ORC have been extensively investigated^3–5^, however, low grade energy has low temperature of heat source, which has resulted in the relatively low efficiency. Therefore, it is of great significance to adopt various means to improve the ORC operation efficiency^6,7^.

Refrigerant is the carrier of energy in thermodynamic cycle, and selecting the appropriate and efficient refrigerant is one of the methods to enhance the ORC operating efficiency. The adsorption and separation of fluid on solid surface are accompanying with the transformation between thermal energy and surface energy^8^; theoretically, such energy transformation is increased with the increase in specific surface area. Consequently, scientists have proposed to add a certain amount of nanoporous particles into the refrigerant to form the nanofluid, so as to modify the thermophysical properties of refrigerant. Chen *et al*.^9^ had extensively investigated the adsorption energy of carbon nano tube (CNT) nanofluid under the action of the thermal, force and electric coupled field. McGrail *et al*.^10^ had carried out simulation study and experimental verification on the adsorption and energy storage of alcohol organic refrigerant in nickel (Ni)-based metal organic framework (MOFs). Porous medium nanofluid shows broad application prospect in the field of energy utilization; nonetheless, study on the adsorption and energy storage of porous medium nanofluid should be further carried out in the face of various refrigerants and porous materials.

There are multiple options regarding the refrigerants of ORC based on the different heat source environments. With the increasing enhancement in people’s environmental awareness, the fourth generation refrigerants with low global warming potential (GWP) and zero ozone depletion potential (ODP) will gradually replace the currently used third generation refrigerants. At present, R1234yf, R1234ze(z) and R32, as well as their mixed refrigerants are one of the current research hotspots^11,12^.

Among the numerous currently available porous materials, MOFs have been extensively recognized as the most promising materials used for adsorption and energy storage^13,14^. MOFs are the porous materials with periodic network structure formed by metal ions or clusters with the organic ligands by means of coordinate self-assembly, which are associated with the advantages of high specific surface area, large porosity, low density and adjustable structure. They have exhibited huge application potentials in the fields of gas storage, separation, heterogeneous catalysis and drug sustained release^15^. Among various MOFs structures, MOF-74 is one of the MOFs with the highest unsaturated metal bit density, and the type of metal ion can be adjusted to modify the adsorption property of MOF-74.

MOFs have nanoscale pore structure, so it is difficult to examine the adsorption property of refrigerant in MOFs through conventional experiment and theoretical method. With the rapid development of computer technology, molecular simulation technique has been extensively applied in scientific research^16–23^. Plenty of literature suggests that, molecular simulation has become the third research means apart from experiment and theory^12,24–29^. Therefore, this study had adopted molecular simulation methods to investigate the adsorption and energy storage properties of R1234yf, R1234ze(z), R134a and R32, as well as their mixtures in M-MOF-74 (M = Mg, Ni).

## Materials and Methods

### Energy storage thermophysical model

The addition of MOFs NPs into the organic refrigerant can obtain the metal organic heat carriers (MOHCs). Theoretically, the energy (∆*h*_MOHCs_) during the endothermic process of MOHCs is mainly constituted by three parts^10,30^, including a. enthalpy of phase change of organic refrigerant (∆*h*_Fluid_); b. thermodynamic energy change ((∫Cpd*T*)_MOFs_) of MOFs particles, and c. desorption heat of fluid refrigerant in MOFs (∆*h*_desorption_), namely,

1 $$\Delta {h}_{{\rm{MOHCs}}}=(1-x)\cdot \Delta {h}_{{\rm{Fluid}}}+x\cdot {(\int {\rm{Cpd}}T)}_{{\rm{MOFs}}}+x\cdot \Delta {h}_{{\rm{desorption}}}$$

Alternatively, it can be expressed as:

2 $$\Delta {h}_{{\rm{MOHCs}}}=\Delta {h}_{{\rm{Fluid}}}+x\cdot ({(\int {{\rm{C}}}_{{\rm{p}}}{\rm{dT}})}_{{\rm{MOFs}}}+\Delta {h}_{{\rm{desorption}}}-\Delta {h}_{{\rm{Fluid}}})$$

where *x* is the mass fraction of MOHC in MOF. It can be discovered from formula (2) that, when the sum of (∫Cpd*T*)_MOFs_ and ∆*h*_desorption_ is greater than ∆*h*_Fluid_, MOHCs can enhance the energy storage property of the refrigerant.

At present, research on the thermophysical property of pure organic refrigerant is relatively mature, which can be obtained through experimental and theoretical methods. In this paper, the thermophysical data of R1234y, R1234ze(z), R134a and R32 were retrieved by the National Institute of Standards and Technology (NIST)^31^. However, MOFs have complex and changeable structures and components, and their adsorption performance and thermophysical property required to be further implemented. In this paper, (∫Cpd*T*)_MOFs_ could be calculated through the thermodynamic energy change^32^ of MOF-74 structure with the increase in temperature obtained through simulation of molecular dynamics (MD). Meanwhile, the desorption heat^33^ of R1234yf, R1234ze(z), R134a and R32, and their mixed refrigerants in MOF-74 would be calculated according to the grand canonical Monte Carlo (GCMC) method. During the GCMC simulations, the chemical potential, volume and temperature of the system are set to be constant value.

### Computational model

**Figure 1:**
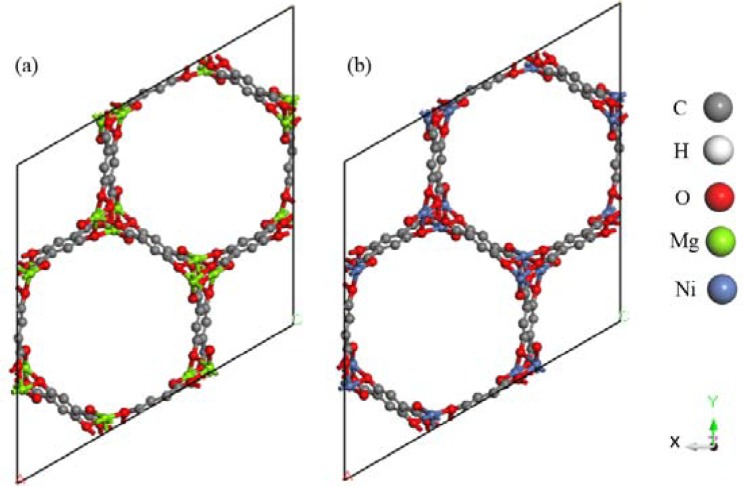
The structure of (**a**) Mg-MOF-74, (**b**) Ni-MOF-74 (1 × 1 × 4 unit cells).

**Figure 2:**
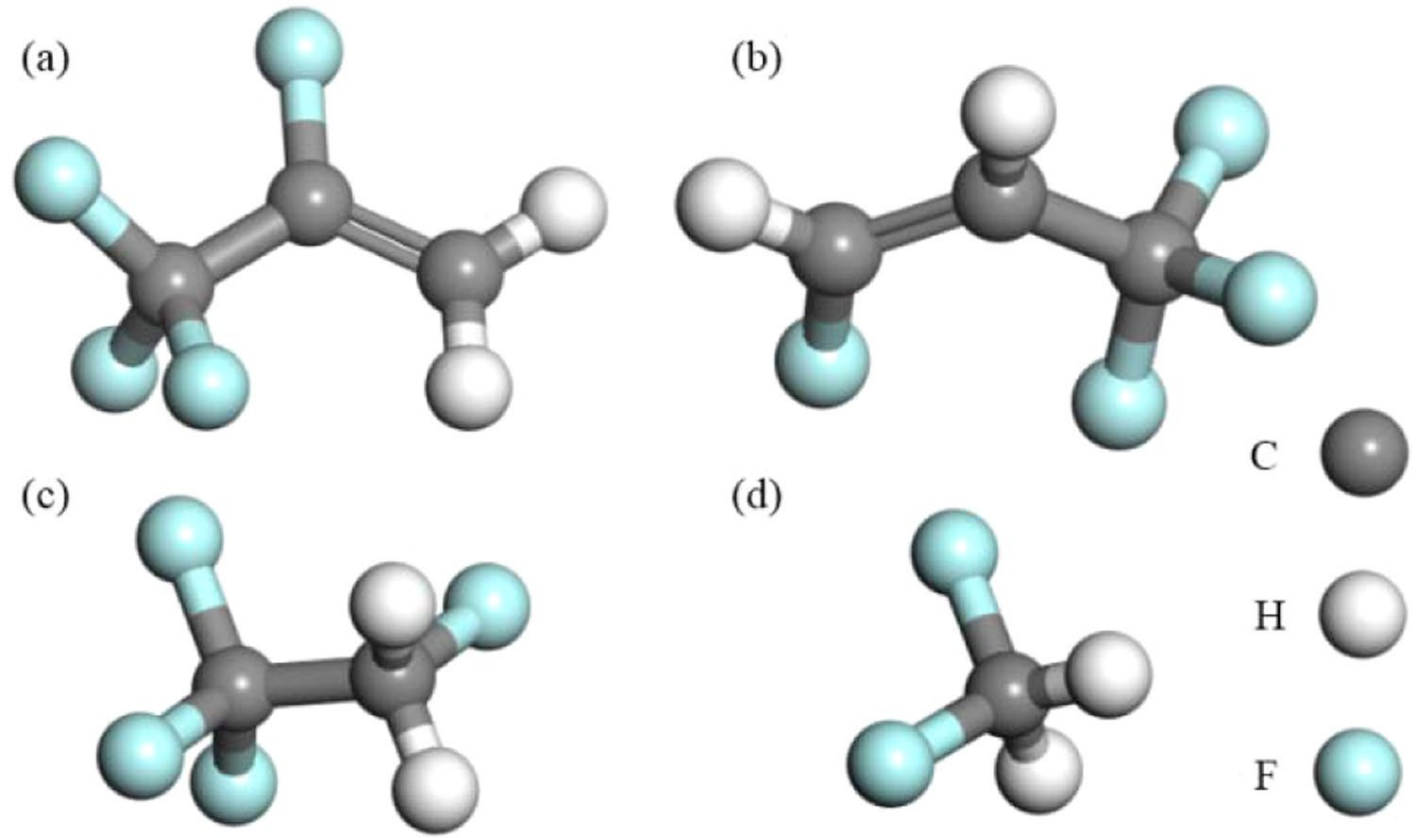
Molecular structures of (**a**) R1234yf, (**b**) R1234ze(z), (**c**) R134a, and (**d**) R32.

The MOFs calculation model in this paper was constituted by the 1 × 1 × 4 M-MOF-74 unit cells. The simulation box was Z: 27.0804 Å in size, and the bottom was an equiside parallelogram with one-side angle of 120 degrees and the side length of 25.7856 Å. M-MOF-74 was constituted by the self-assembly of divalent metal ions with the ligand 2,5-dihydroxyl terephthalic acid, which had formed the 3D alveolate spatial network structure with 2D hexogonal channel, and the metal atoms were formed in the manner of octahedral coordination (occupied by 5 oxygen atoms and 1 water molecule). There were 648 atoms in the model, including 288 C, 72 H, 216 O, and 72 Mg/Ni, as presented in Fig. 1. The molecular configuration of R1234yf, R1234ze(z), R134a and R32 is shown in Fig. 2.

The MD and GCMC simulations in this paper were realized on the Materials Studio molecular simulation software^34,35^, which offers a rich set of features for materials modeling. In the simulation, MD and GCMC had adopted the COMPASS force field^36,37^ to describe the inter-atom interactions, the Ewald method was employed to manage the long-range electrostatic interaction between particles, and the periodic boundary conditions were applied in the X, Y and Z directions of the simulation box^38–40^. The cut off radius is 10 Å in the simulations.

### MD Simulation details

The Forcite module in Materials Studio was selected for MD simulation, and the thermodynamic energy changes of MOF-74 particles under different temperatures were calculated in the NVT system. The time step was set at 1 *fs*. The systems were run 1,000 *ps* to reach the equilibrate state first. Then, system run another 1,000 ps to analysis the data with information of atoms stored every 1,000 *fs* to analyze the results. The calculation temperatures were selected at 293 K, 313 K, 333 K, 353 K, 373 K and 393 K. The Berendsen thermostat heat bath method was employed to control the system temperature^41^. All the MD simulation work repeats three times. The uncertainties of the MD simulations is the statistical error of the data with information of atoms stored every 1,000 *fs* after the balance.

### GCMC Simulation Details

The Sorption module in Materials Studio was selected for GCMC simulation, so as to calculate the isothermal adsorption processes of R1234yf, R1234ze(z), R134a, R32, and their mixed refrigerants in MOF-74 at different temperatures (293K, 313K, 333K, 353K, 373K and 393K). Among them, the pure refrigerant simulation pressure range was 1-5,000 kPa, while that of mixed refrigerants (R1234yf/R32, R1234ze(z)/R32, R1234yf/R134a, R1234ze(z)/R134a) was 1-16,000 kPa. The fugacity was calculated using the Peng-Robinson equation. Each state point was balanced after 1,000,000 cycles and averaged with 2,000,000 cycles. The data with information of atoms stored every 2,000 cycles to analyze the results. And the uncertainties of the GCMC simulation is the data with information of atoms stored every 2,000 cycles after the balance. The uncertainties are shown as error bars in Fig.

**Figure 3:**
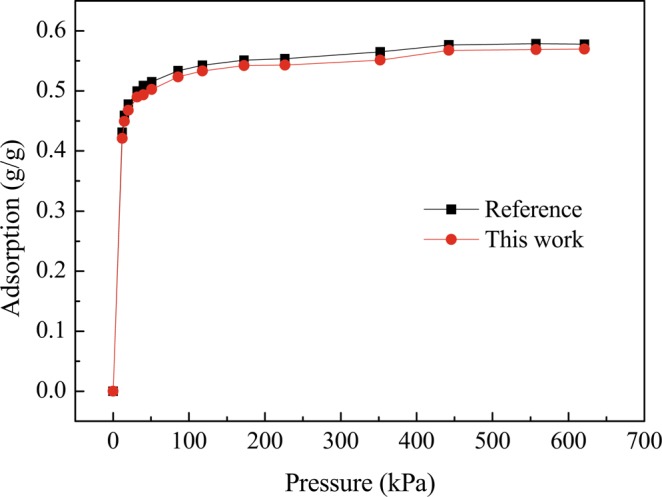
Adsorption of R134a in M-MOF-74 at 298K (The black and read curves correspond to reference work and this simulation work, respectively.).

## Results and Discussions

In order to check the reliability of the simulation work, the adsorption of R134a in M-MOF-74 was simulated at 298K and compared with the experimental work of Zheng *et al*.^42,43^, as shown in Fig. 3. It would better reveal the reliability of the simulation work in this investigation to some extent.

### Pure refrigerant adsorption

**Figure 4:**
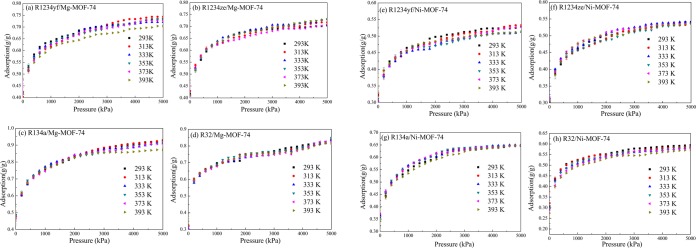
Adsorption of R1234yf, R1234ze(z), R134a and R32 in M-MOF-74. (**a**–**d**) Adsorption of R1234yf, R1234ze(z), R134a and R32 in Mg-MOF-74, respectively; (**e**–**h**) Adsorption of R1234yf, R1234ze(z), R134a and R32 in Ni-MOF-74, respectively.

Herein, Fig. 4 presents the adsorption isotherms of R1234yf, R1234ze(z), R134a and R32 pure refrigerants in M-MOF-74 at 6 calculation temperatures. It could be discovered that, the adsorption quantities of R134a and R32 in M-MOF-74 were slightly higher than those of R1234yf and R1234ze(z) in corresponding M-MOF-74. This was mainly related to the molecular structure size. As shown in Fig. 2, the molecular sizes of R134a and R32 were smaller than those of R1234yf and R1234ze(z), thus more R134a and R32 were adsorbed into the MOF-74 pore. Additionally, the adsorption quantities of R1234yf, R1234ze(z), R134a and R32 in Mg-MOF-74 were greater than those in Ni-MOF-74, which was because that the ion radius of Mg^2+^ was smaller than that of Ni^2+^, so that Mg-MOF-74 had stronger attracting effect^44,45^.

**Figure 5:**
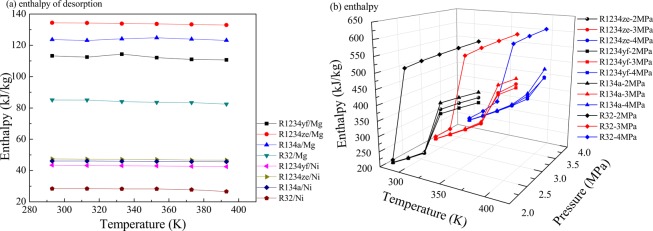
(**a**) Desorption heat of R1234yf, R1234ze(z), R134a and R32 in M-MOF-74 and Ni-MOF-74 under saturation state; (**b**) enthalpy values of R1234yf, R1234ze(z), R134a and R32 at 2 MPa, 3 MPa and 4 MPa, respectively.

In order to calculate the energy difference (∆*h*_MOHCs_), the desorption heat and enthalpy values of all the refrigerants are calculated. Figure 5 has displayed the enthalpy values of R1234yf, R1234ze(z), R134a and R32 under different pressures retrieved through NIST, as well as the desorption heat of R1234yf, R1234ze(z), R134a and R32 in M-MOF-74 calculated by GCMC. At saturation adsorption, the desorption heat of R32 in MOF-74 was smaller than that of R1234yf, R1234ze(z) and R134a in MOF-74. The desorption heat of R1234yf, R1234ze(z), R134a and R32 in Mg-MOF-74 was greater than that in Ni-MOF-74, which was because that, Mg-MOF-74 had greater adsorption capacity than Ni-MOF-74. However, the enthalpies of phase change of R32 within the researched temperature and pressure ranges were greater than those of R1234yf, R1234ze(z) and R134a.

### Adsorption and energy storage of pure refrigerant

It had been proved in previous work^46^ that, the MD method could obtain reliable thermodynamic energy changes. In this paper, MD calculation could obtain the thermodynamic energy values of M-MOF-74 particles under different temperatures, as shown in Table 1. The thermodynamic energy value of MOF-74 particles was increased with the increase in temperature, which conformed to the general variation rule of solid material under heating. The Cp represented the slope of curve of thermodynamic energy with temperature, and the calculated values in this paper were consistent with those reported in literature^44^.

**Table 1:** Thermodynamic energy of M-MOF-74.

Temperature (K)	293	313	333	353	373	393
Thermodynamic energy (kJ/kg)	**Mg-MOF-74**					
	−21909.3 ±30.2	−21829.3 ±35.1	−21749.3 ±33.5	−21669.4 ±40.7	−21589.4 ±42.8	−21509.4 ±38.6
	**Ni-MOF-74**					
	−895.9 ± 14.8	−860.0 ± 12.5	−824.1 ± 14.1	−788.2 ± 13.3	−752.3 ± 10.7	−716.4 ± 15.5

As mentioned in section 2.1 above, the energy storage of MOHCs at the time of heat adsorption could be calculated by formula (1) or formula (2). Among them, the enthalpy of phase change of organic refrigerant (∆*h*_Fluid_) and the desorption heat of fluid refrigerant in MOFs (∆*h*_desorption_) were calculated from Fig. 5(a,b), respectively. And the thermodynamic energy change ((∫Cpd*T*)_MOFs_) of MOFs particles was calculated from Table 1. Additionally, the energy difference (∆*h*_MOHCs_) can be calculated by the three parts of ∆*h*_Fluid_, ∆*h*_desorption_ and (∫Cpd*T*)_MOFs_. After that, the energy storage enhancement (∆*E*_enhancement_) of MOHCs was calculated by,

3 $$\varDelta {E}_{{\rm{enhancement}}}=\frac{\varDelta {h}_{{\rm{MOHCs}}}-\varDelta {h}_{{\rm{MOHCs}},x=0,{\rm{293K}}}}{\varDelta {h}_{{\rm{MOHCs}},x=0,{\rm{293K}}}}$$

where ∆*E*_enhancement_ represents the increase in energy storage. ∆*h*_MOHCs,*x*=0,293K_ is the energy difference (∆*h*_MOHCs_) of the discussed refrigerant with the mass fraction of MOHCs *x* = 0 at 293 K.

**Figure 6:**
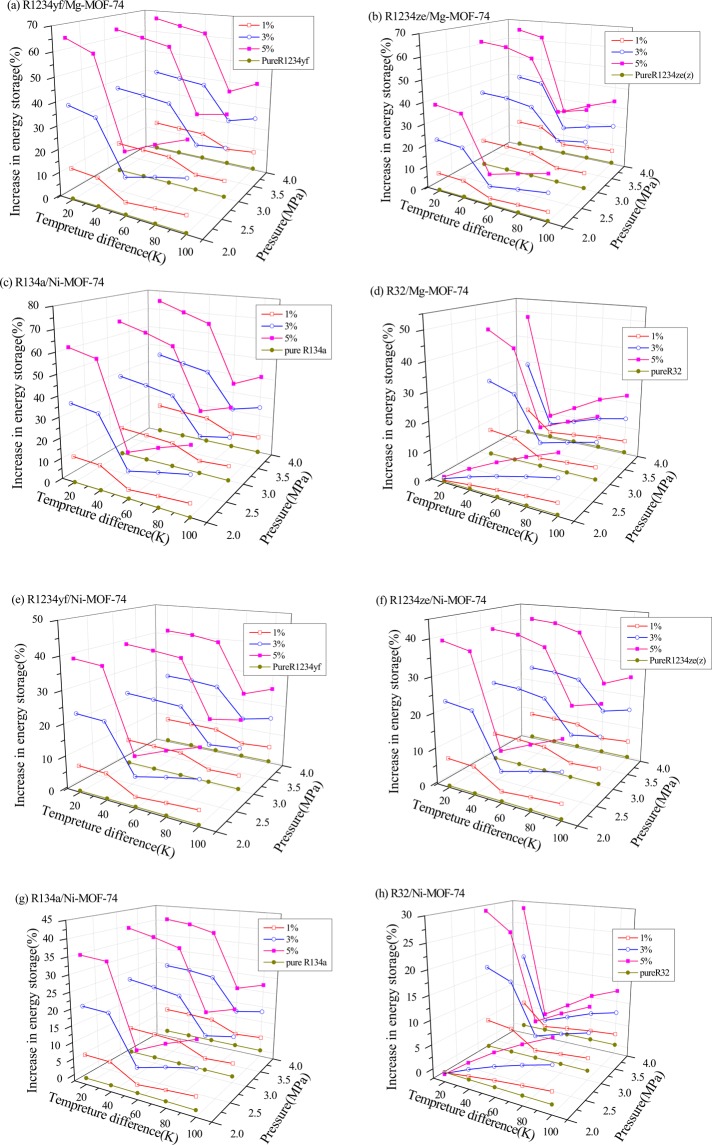
The energy storage enhancement of pure refrigerants R1234yf, R1234ze(z), R134a and R32 in M-MOF-7 with the temperature difference at 2 MPa, 3 MPa and 4 MPa.

Figure 6 demonstrates the relationship of energy storage enhancement of pure refrigerants R1234yf, R1234ze(z), R134a and R32 in M-MOF-74 particles at different mass fractions and temperatures during the heat adsorption process, which calculated at the reference pressures (2 MPa, 3 Mpa and 4 MPa). It could be discovered in Fig. 6 that, the addition of M-MOF-74 NPs in the pure refrigerants R1234yf, R1234ze(z), R134a and R32 could enhance the energy storage properties of refrigerants; besides, the energy storage effect was enhanced with the increase in the mass fraction of M-MOF-74 NPs. R1234yf shared similar molecular structure with R1234ze(z), and the M-MOF-74 NPs showed almost consistent effect on enhancing the heat storage of R1234yf and R1234ze(z), which was superior to that on R32, but was lower than that on R134a. Moreover, in the MOHCs property curve constituted by R32 and Ni-MOF-74, negative enhancement effect occurred at the temperature difference of 20 K, as presented in Fig. 6(h). This was mainly because that R32 had a too large enthalpy of phase change ∆*h*_Fluid_, which had surpassed the sum of the variation value of Ni-MOF-74 thermodynamic energy (∫C_p_d*T*)_MOFs_ with temperature and the desorption heat of R32 ∆*h*_desorption_ in Ni-MOF-74.

### Adsorption of mixed refrigerants

**Figure 7:**
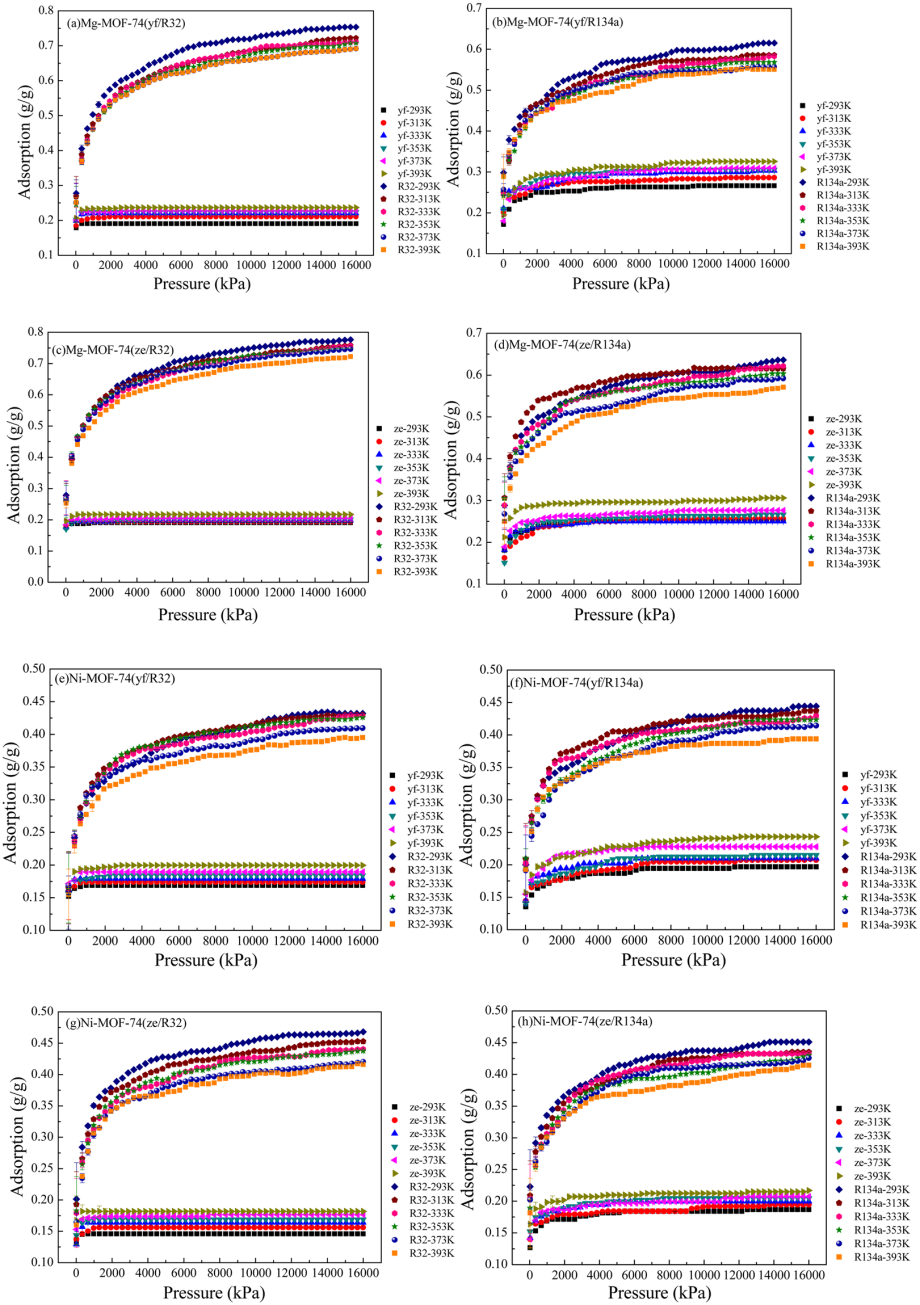
Competitive adsorption of mixed refrigerants R1234yf/R32, R1234yf/R134a, R1234ze(z)/R32 and R1234ze(z)/R134a in M-MOF-74. (**a**–**d**) Adsorption of R1234yf/R32, R1234yf/R134a, R1234ze(z)/R32 and R1234ze(z)/R134a in Mg-MOF-74, respectively; (**e**–**h**)Adsorption of R1234yf/R32, R1234yf/R134a, R1234ze(z)/R32 and R1234ze(z)/R134a in Ni-MOF-74, respectively.

Herein, Fig. 7 shows the competitive adsorption behaviors of mixed refrigerants R1234yf/R32, R1234yf/R134a, R1234ze(z)/R32 and R1234ze(z)/R134a in M-MOF-74 under different temperatures and pressures. The adsorption quantities of R32 and R134a were far higher than those of R1234yf and R1234ze(z); besides, the adsorption quantities of R32 and R134a were reduced with the increase in temperature, while those of R1234yf and R1234ze(z) were increased with the increase in temperature. Compared with R32 and R134a, R1234yf and R1234ze(z) could reach the saturation adsorption status in a faster rate, which was because that the small molecule structures of R32 and R134a could effectively utilize the pore structure in MOF-74 to increase the adsorption quantity. With the increase in temperature, the molecular thermal motion was enhanced, some R32 could be desorbed from MOF-74, the empty points within MOFs were increased, and a small amount of R1234yf and R1234ze(z) would enter into the MOFs for adsorption. At the same time, in the mixed refrigerant adsorption, the adsorption quantity of each refrigerant was lower than that of pure refrigerant, which was because that the combined adsorption of mixed refrigerants would occupy the pore space in MOFs. On the other hand, the adsorption quantities of R1234yf and R1234ze(z) in the R1234yf/R134a and R1234ze(z)/R134a mixtures under the same temperature and pressure were greater than those of R1234yf/R32 and R1234ze(z)/R32. In the meantime, the adsorption quantities of R1234yf, R1234ze(z), R134a and R32 in Mg-MOF-74 in the mixture were greater than those in Ni-MOF-74.

**Figure 8:**
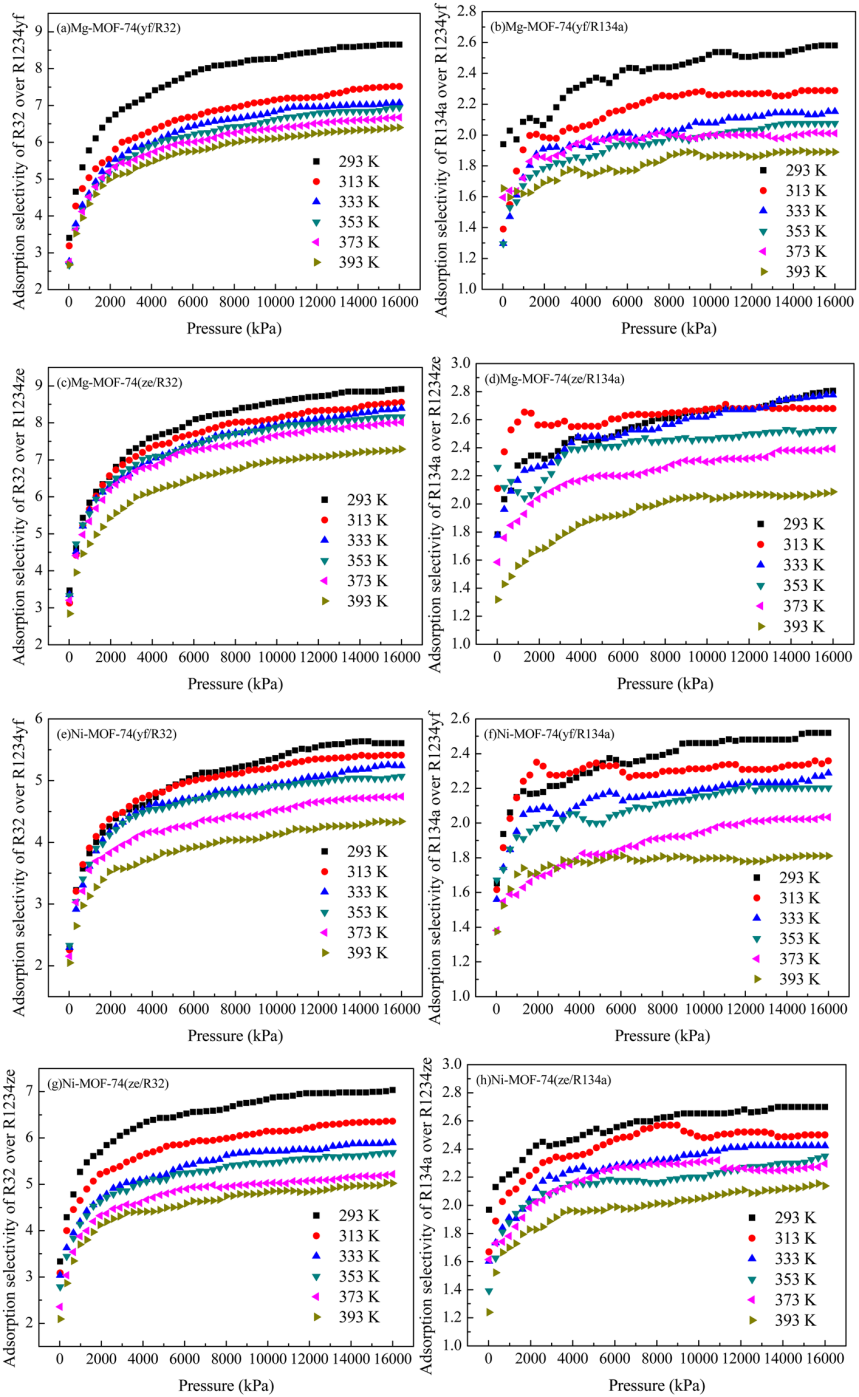
Adsorption selectivity of R32/R134a over R1234yf/ R1234ze(z) in M-MOF-74. (**a**–**d)** adsorption in Mg-MOF-74; (**e**–**h**) adsorption in Ni-MOF-74. (**a**,**e**) adsorption selectivity of R32 over R1234yf; (**b**,**f**) adsorption selectivity of R134a over R1234yf; (**c**,**g**) adsorption selectivity of R32 over R1234ze(z); (**d**,**h**) adsorption selectivity of R134a over R1234ze(z).

Then the adsorption selectivity was calculated to assess the adsorption capacity of a sorbent in mixture competitive adsorption simulations. The adsorption selectivity of sorbent A over sorbent B in M-MOF-74 was defined as,

4 $${S}_{{\rm{A}}}=\frac{{x}_{{\rm{A}}}/{y}_{{\rm{A}}}}{{x}_{{\rm{B}}}/{y}_{{\rm{B}}}}$$

where *x*_*i*_ and *y*_*i*_ are the mole fraction of sorbent *i* in the adsorbed phase and bulk phase, respectively. The adsorption selectivity of R32/R134a over R1234yf/R1234ze(z) in M-MOF-74 was calculated and the results were shown in Fig. 8. All selectivity values were greater than one, which indicated that the adsorption capacity of R32/R134a was superior to R1234yf/R1234ze(z) in M-MOF-74. Additionally, this superiority would decrease with increasing temperature. The adsorption selectivity of R32 over R1234ze(z) was the maximum value among the binary adsorptions in both MOF-74. Thereafter, the adsorption selectivity of R32 over R1234ze(z) in Mg-MOF-74 was higher than that in Ni-MOF-74, which indicated that Mg-MOF-74 had better adsorption selectivity than that of Ni-MOF-74.

**Figure 9:**
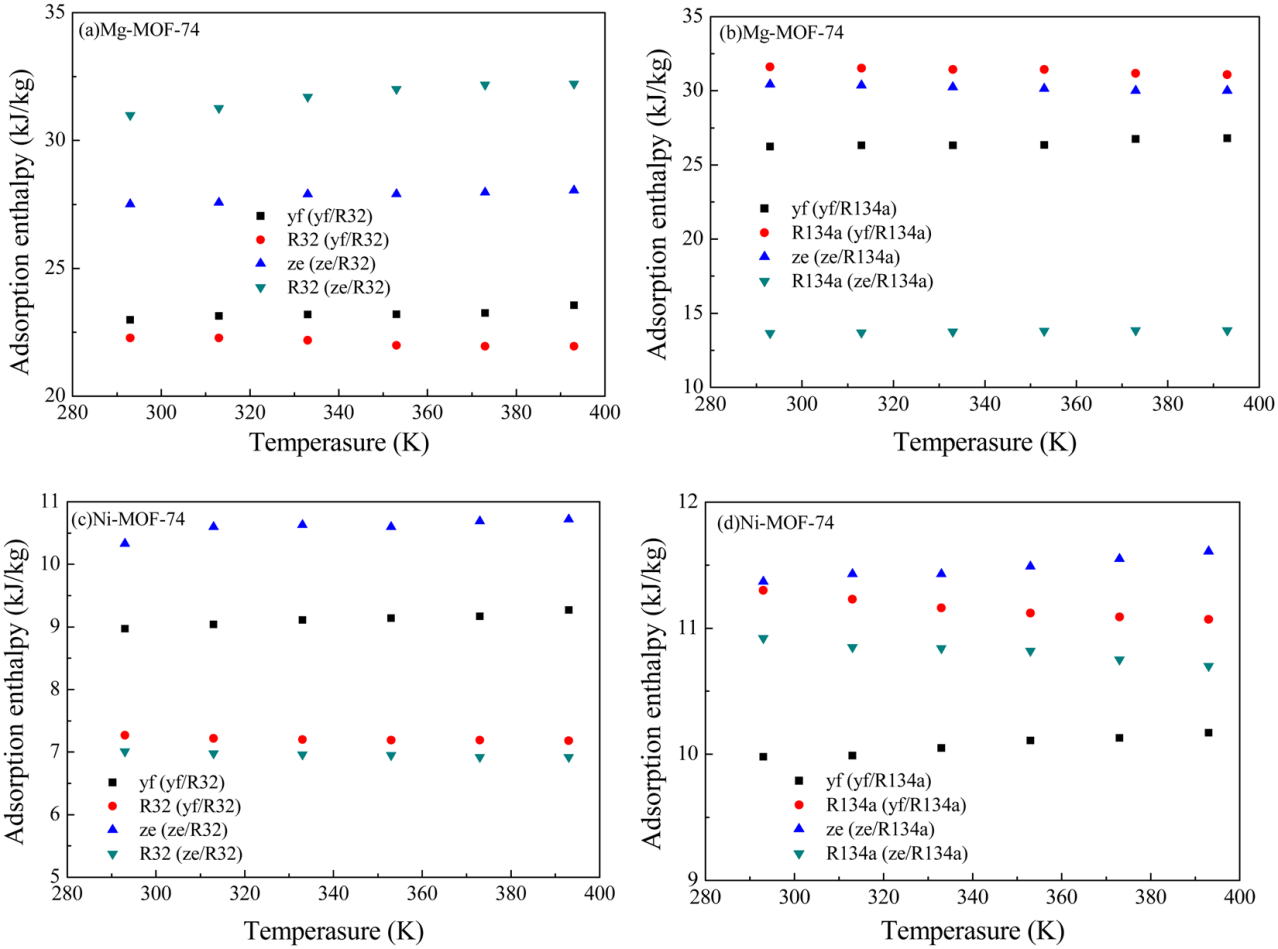
Enthaplies of R1234yf/R32, R1234yf/R134a, R1234ze(z)/R32 and R1234ze(z)/R134a in competitive adsorption in M-MOF-74.

Figure 9 shows the adsorption enthalpies of mixed refrigerants in M-MOF-74 under different temperatures calculated through GCMC. In Mg-MOF-74, the desorption enthalpy of R1234ze(z)/R32 was higher than that of R1234yf/R32, while that of R1234ze(z)/R134a was lower than that of R1234yf/R134a. The desorption enthalpies of the mixed refrigerants in Mg-MOF-74 were higher than those in Ni-MOF-74.

## Conclusions

The efficiency of thermodynamic cycles can be improved by using the optimized working fluids. Using MD and GCMC simulation methods, this paper has studied the adsorption and energy storage performance of R1234yf, R1234ze(z), R32 and their mixed refrigerants in Mg-MOF-74 and Ni-MOF-74. The adsorption quantities of R32 and R134a in MOF-74 are higher than those of R1234yf and R1234ze(z), while at saturation adsorption state, the desorption heat of R32 in MOF-74 is lower than that of R1234yf and R1234ze(z). The adsorption quantities and desorption enthalpies of refrigerants in Mg-MOF-74 are higher than those in Ni-MOF-74. Noteworthily, the addition of MOF-74 NPs in the pure refrigerant can enhance its energy storage capacity; besides, the addition of MOF-74 NPs in R1234yf and R1234ze(z) to form MOHCs can result in superior heat storage enhancement effect to that of R32 MOHCs. Furthermore, in mixed refrigerant competitive adsorption, the adsorption quantities of R1234ze(z) and R1234yf are lower than those of R32 and R134a; with the increase in temperature, the adsorption quantities of R1234ze(z) and R1234yf are gradually increased, while those of R32 and R134a are gradually decreased. And Mg-MOF-74 has better adsorption selectivity than that of Ni-MOF-74. In general, this study is of great significance for theoretical analysis of adsorption characteristics of refrigerant molecules in porous media. And it can provide reliable basis for selecting appropriate refrigerant working mediums and MOFs to form optimal MOHCs in engineering.

## References

[1] Zhang C, Liu C, Wang S, Xu X, Li Q (2017). Thermo-economic comparison of subcritical organic Rankine cycle based on different heat exchanger configurations. Energy.

[2] Shen H (2019). A Novel TiZrHfMoNb High-Entropy Alloy for Solar Thermal Energy Storage. Nanomaterials.

[3] Liu T (2019). Operation Characteristics and Transient Simulation of an ICE-ORC Combined System. Appl. Sci.

[4] Yamamoto T, Furuhata T, Arai N, Mori K (2001). Design and testing of the organic rankine cycle. Energy.

[5] Dai Y, Wang J, Gao L (2009). Parametric optimization and comparative study of organic rankine cycle (ORC) for low grade waste heat recovery. Energy Conv. Manag.

[6] Andwari AM, Pesyridis A, Esfahanian V, Salavati-Zadeh A, Hajialimohammadi A (2019). Modelling and Evaluation of Waste Heat Recovery Systems in the Case of a Heavy-Duty Diesel Engine. Energies.

[7] Ding Y (2018). Exergoenvironmental model of Organic Rankine Cycle system including the manufacture and leakage of working fluid. Energy.

[8] Li Q, Liu C (2012). Molecular dynamics simulation of heat transfer with effects of fluid–lattice interactions. Int. J. Heat Mass Transf.

[9] Chen X, Xu B, Liu L (2014). Nanoscale Fluid Mechanics and Energy Conversion. Appl. Mech. Rev.

[10] McGrail BP (2013). Metal-organic heat carrier nanofluids. Nano Energy.

[11] Yataganbaba A, Kilicarslan A, Kurtbaş İ (2015). Exergy analysis of R1234yf and R1234ze as R134a replacements in a two evaporator vapour compression refrigeration system. Int. J. Refrig..

[12] Raabe G (2013). Molecular Simulation Studies on the Vapor–Liquid Phase Equilibria of Binary Mixtures of R-1234yf and R-1234ze(E) with R-32 and CO_2_. J. Chem. Eng. Data.

[13] Li JR, Sculley J, Zhou HC (2012). Metal–organic frameworks for separations. Chem. Rev..

[14] Zhou Y, Li Q, Wang Q (2019). Energy Storage Analysis of UIO-66 and Water Mixed Nanofluids: An Experimental and Theoretical Study. Energies.

[15] Furukawa H, Cordova KE, O’Keeffe M, Yaghi OM (2013). The chemistry and applications of metal-organic frameworks. Science.

[16] Li Q, Liu C, Chen X (2014). Molecular dynamics simulation of sulphur nucleation in S–H2S system. Mol. Phys..

[17] Peng T, Li Q, Liu C (2016). Accelerated aqueous nano-film rupture and evaporation induced by electric field: A molecular dynamics approach. Int. J. Heat Mass Transf.

[18] Alam MS, Jeong JH (2018). Molecular dynamics simulations on homogeneous condensation of R600a refrigerant. J. Mol. Liq..

[19] Liu Y, Chen X (2013). High permeability and salt rejection reverse osmosis by a zeolite nano-membrane. Phys. Chem. Chem. Phys..

[20] Cai, S., Tang, Q., Tian, S., Lu, Y. & Gao, X. Molecular Simulation Study on the Microscopic Structure and Mechanical Property of Defect-Containing sI Methane Hydrate, *Int. J. Mol. Sci.* **20**, 2305 (2019). 10.3390/ijms20092305 PMC6539317 31075976

[21] Wang Z, Gu T, Kadohira T, Tada T, Watanabe S (2008). Migration of Ag in low-temperature Ag(2)S from first principles. J. Phys. Chem..

[22] Fu T (2015). Molecular dynamics simulation of VN thin films under indentation. Appl. Surf. Sci..

[23] Fu T (2017). Molecular dynamics simulation of plasticity in VN(001) crystals under nanoindentation with a spherical indenter. Appl. Surf. Sci..

[24] Frenkel, D. & Smit, B. Understanding Molecular Simulation: from Algorithms to Applications (Second Edition). New York: Academic Press (2002).

[25] Peguin RPS, Kamath G, Potoff JJ, da Rocha SRP (2009). All-Atom Force Field for the Prediction of Vapor-Liquid Equilibria and Interfacial Properties of HFA134a. J. Phys. Chem. B.

[26] Stoll J, Vrabec J, Hasse H (2003). A set of molecular models for carbon monoxide and halogenated hydrocarbons. J. Chem. Phys..

[27] Zhang, L., Tian, S. & Peng, T. Molecular Simulations of Sputtering Preparation and Transformation of Surface Properties of Au/Cu Alloy Coatings Under Different Incident Energies, *Metals* **9**, 259 (2019).

[28] Fu T (2016). Molecular dynamics simulation of nanoindentation on Cu/Ni nanotwinned multilayer films using a spherical indenter. Sci. Reports.

[29] Fu T (2016). Molecular dynamics simulation of effects of twin interfaces on Cu/Ni multilayers. Mater. Sci. Eng.

[30] Li Q, Xiao Y, Shi X, Song S (2017). Rapid Evaporation of Water on Graphene/Graphene-Oxide: A Molecular Dynamics Study. Nanomaterials.

[31] NIST, http://webbook.nist.gov/chemistry/fluid/.

[32] Li Q (2017). Molecular dynamics simulations of aggregation of copper nanoparticles with different heating rates. Physica E.

[33] Lei G, Liu C, Li Q, Xu X (2016). Graphyne nanostructure as a potential adsorbent for separation of H2S/CH4 mixture: Combining grand canonical Monte Carlo simulations with ideal adsorbed solution theory. Fuel.

[34] Accelrys, I. Materials Studio. Accelrys Software Inc. (2010).

[35] Purse M (2009). Reactive Molecular Dynamics Study of the Thermal Decomposition of Phenolic Resins. J. Compos. Sci..

[36] Rappe AK, Casewit CJ, Colwell KS, Goddard WA, Skiff WM (1992). UFF, a full periodic table force field for molecular mechanics and molecular dynamics simulations. J. Am. Chem. Soc..

[37] De Lorenzo L, Tocci E, Gugliuzza A, Drioli E (2012). Pure and Modified Co-Poly (amide-12-b-ethylene oxide) Membranes for Gas Separation Studied by Molecular Investigations. Membranes.

[38] Xiang H (2018). Molecular dynamics simulation for orientation dependence of deformations in monocrystalline AlN during nanoindentation. Ceram. Int..

[39] Hu H, Weinberger CR, Sun Y (2014). Effect of Nanostructures on the Meniscus Shape and Disjoining Pressure of Ultrathin Liquid Film. Nano Lett..

[40] Rao Z, Wang S, Peng F (2013). Molecular dynamics simulations of nano-encapsulated and nanoparticle-enhanced thermal energy storage phase change materials. Int. J. Heat Mass Transf..

[41] Lemak AS, Balabaev NK (2006). On The Berendsen Thermostat. Mol. Simul..

[42] Zheng J (2020). Molecular Insight into Fluorocarbon Adsorption in Pore Expanded Metal−Organic Framework Analogs. J. Am. Chem. Soc..

[43] Zheng J (2017). Pore-Engineered Metal−Organic Frameworks with Excellent Adsorption of Water and Fluorocarbon Refrigerant for Cooling Applications. J. Am. Chem. Soc..

[44] Hu J, Liu C, Liu L, Li Q (2018). Thermal Energy Storage of R1234yf, R1234ze, R134a and R32/MOF-74 Nanofluids: A Molecular Simulation Study. Materials.

[45] Pham T (2014). Understanding the H2 Sorption Trends in the M-MOF-74 Series (M = Mg, Ni, Co, Zn). J. Phys. Chem. C.

[46] Hu J, Liu C, Li Q, Shi X (2018). Molecular simulation of thermal energy storage of mixed CO2/IRMOF-1 nanoparticle nanofluid. Int. J. Heat Mass Transf..

