# Author Correction: Molecular Simulations of Adsorption and Energy Storage of R1234yf, R1234ze(z), R134a, R32, and their Mixtures in M-MOF-74 (M = Mg, Ni) Nanoparticles

**DOI:** 10.1038/s41598-020-74320-5

**Published:** 2020-10-08

**Authors:** Shouyin Cai, Sen Tian, Yiyu Lu, Guangjin Wang, Yu Pu, Kang Peng

**Affiliations:** 1 Key Laboratory of Low-grade Energy Utilization Technologies & Systems, Ministry of Education, College of Energy and Power Engineering, Chongqing University, Chongqing, 400044 People’s Republic of China; 2 State Key Laboratory of Coal Mine Disaster Dynamics and Control, School of Resources and Safety Engineering, Chongqing University, Chongqing, 400044 People’s Republic of China; 3 Yunnan Key Laboratory of Sino-German Blue Mining and Utilization of Special Underground Space, Faculty of Land Resources Engineering, Kunming University of Science and Technology, Kunming, 650093 People’s Republic of China

Correction to: *Scientific Reports*. 10.1038/s41598-020-64187-x, published on 29 April 2020

The original version of this Article contained a typographical error in the spelling of the author Yiyu Lu, which was incorrectly given as Yuyi Lu. This has now been corrected in the PDF and HTML versions of the Article.

